# The RAPID3 questionnaire as a screening tool to reduce the number of outpatient clinic visits: a retrospective cohort study

**DOI:** 10.1007/s10067-022-06162-7

**Published:** 2022-04-26

**Authors:** J. Wiegel, B. F. Seppen, M. M. ter Wee, M. T. Nurmohamed, M. Boers, W. H. Bos

**Affiliations:** 1 Amsterdam Rheumatology & Immunology Center, Reade, Amsterdam, the Netherlands; 2 Amsterdam Rheumatology & Immunology Center, Amsterdam UMC Location VUmc, Amsterdam, the Netherlands; 3 Department of Epidemiology & Data Science, Amsterdam Public Health, Amsterdam UMC Location VUmc, Amsterdam, the Netherlands

**Keywords:** DAS28, Patient-reported outcomes, RAPID3, Rheumatoid arthritis

## Abstract

**Background:**

Treat-to-target strategies require frequent on-site evaluations of disease activity in patients with rheumatoid arthritis (RA), burdening patients and caregivers. However, this frequency may not be required in patients in a stable low disease activity state. The Routine Assessment of Patient Index Data 3 (RAPID3) is a reliable tool to detect such states in groups but has not been tested to reduce the frequency of on-site evaluations in individual patient care. In Reade, an outpatient rheumatology clinic, patients can complete the questionnaire online prior to consultation, and the results are directly fed into the electronic patient record. Focusing on low disease activity, we retrospectively studied the test characteristics of RAPID3 and its agreement with the DAS28 in our database of routine patient care.

**Objective:**

To assess the test characteristics and agreement between de DAS28 and the RAPID3 in patients with RA, with a focus on the low disease activity categories.

**Methods:**

We performed a retrospective database study with available clinical data collected as part of usual care from the electronic medical record at Reade Amsterdam. The dataset comprised RAPID3 assessments followed by a DAS28 within 2 weeks, obtained between June 2014 and March 2021. We dichotomized the disease activity categories for both the RAPID3 and DAS28 into low (remission and low disease activity) and high (moderate and high disease activity). With cutoff values of 2.0 for RAPID3 and 3.2 for DAS28, we calculated test characteristics and agreement (Cohen’s kappa).

**Results:**

A total of 5009 combined RAPID3 and DAS28 measurements were done at Reade in 1681 unique RA patients. The mean age was 60 years, and 76% of patients were female with a median disease duration of 4 years. Agreement was considered fair (kappa = 0.26). In total, 1426 (28%) of the RAPID3 measurements were classified as low and could be potentially targeted to skip their consultations. The sensitivity to detect low disease activity was 0.39, specificity was 0.93, and the positive predictive value was 0.92.

**Conclusion:**

We showed that when the RAPID3 classifies a patient into low disease activity state, the accuracy is 92%. Of all consultations, 28% could possibly be postponed following the screening with RAPID3.

**Table:** 

**Key Points** *• Most studies conclude that the RAPID3 alone is insufficient to monitor the disease activity of RA due to its general overestimation of the disease activity compared with the DAS28.* *• Our results show that in 92% of the cases patients with a RAPID3 ≤ 2.0 have a DAS28 ≤ 3.2.* *• We propose a system where the RAPID3 is used to screen for patients in remission/low disease activity, in order to postpone consultations of these patients and reduce the number of unnecessary outpatient clinic visits.*	

## Introduction

Rheumatoid arthritis (RA) is a chronic inflammatory disease characterized by joint inflammation. RA has a variable course where flares of disease activity alternate with episodes of low disease activity. Guidelines advise close monitoring of disease activity and treating-to-target to minimize and inhibit radiologic destruction of joints [1]. Patients are therefore chronically monitored 2–4 times a year, leading to a large number of protocolized outpatient clinic visits. However, most of these visits could probably be postponed or even omitted, as 75% of patients in routine clinical follow-up are in low disease activity or remission [2]. Reducing the number of unnecessary outpatient clinic visits will improve access for patients in need of rapid consultation and reduce healthcare and patient costs. This reduction in visits is essential due to a growing demand for rheumatology healthcare, driven by the overall increase in healthcare utilization and the increasing number of patients with RA. It is estimated that this will lead to a significant shortage of rheumatologists in 2030, where more healthcare needs to be provided with the same capacity of people and resources [3].

The development of electronic patient-reported outcomes (ePROs) that assess disease activity in rheumatic care has created the opportunity to monitor patients at home [4]. Traditional disease activity scores such as the disease activity score 28 (DAS28) and the simple- and clinical disease activity index (SDAI resp. CDAI) require physical examination (joint counts), and DAS28 and SDAI also laboratory testing at the outpatient clinic, unlike PROs scored by patients [5, 6]. Thus, ePRO’s can estimate disease activity prior to consultation, and consultations could be postponed in case of low disease activity. However, misclassification would lead to unjustified postponements and suboptimal care. So screening for low disease activity with a PRO needs to be safe (low number of unjustified postponements) and effective (high number of justified postponements).

In routine care, the DAS28 and the Routine Assessment of Patient Index Data 3 (RAPID3) are frequently used instruments. The DAS28 is an index of painful and tender joint counts, patient global assessment, and acute phase reactant [5]. The RAPID3 is a PRO-derived index of physical disability, pain, and patient global assessment [6]. Both can classify patients into four disease activity states: “remission,” low, moderate, or high disease activity. So far, research comparing the RAPID3 with the DAS28 has focussed mainly on their correlation or on their agreement in identifying flares. With correlation coefficients ranging from 0.5 to 0.9 and contradicting reports on the agreement in the different disease activity categories, the RAPID3 alone appears to be insufficient to follow long-term disease activity in patients with RA in clinical practice [7–15]. However, the ability of the RAPID3 to screen for patients in remission or low disease activity has not been studied.

Our aim was to assess the test characteristics and agreement between de DAS28 and the RAPID3 in patients with RA, with a focus on the low disease activity categories.

## Methods

### Setting and patient population

We performed a retrospective database study with available clinical data from the electronic medical record (EMR) at Reade Amsterdam. Reade has a large outpatient clinic for rheumatology patients in Amsterdam. Since June 2014, each patient is requested by email to complete the RAPID3 before their consultation at the clinic. We extracted the database from the EMR comprising each completed RAPID3 between June 2014 and March 2021, which was followed by a DAS28 within 2 weeks in patients with RA. Regular DAS28 and RAPID3 measurements are part of usual care at Reade (following EULAR guidelines for the management of RA [16]). We selected patients diagnosed with RA according to the ICD-10 criteria M06.99 (RA, unspecified), M06.09 (RA, without rheumatoid factor), or M05.99 (RA, seropositive). We collected additional information such as sex, age, and disease duration.

### Disease activity measures

This study evaluated the accuracy of the RAPID3 to detect DAS28 low disease activity or remission and its accuracy to detect ACR-EULAR Boolean remission as a secondary outcome [17]. The RAPID3 score uses the following thresholds: remission 0–1.0, low 1.1–2.0, moderate 2.1–4.0, and high disease activity > 4.0 [11]. The DAS28 uses the same terms for disease activity classes, with cut thresholds for remission < 2.6, low 2.6–3.1, moderate 3.2–5.1, and high disease activity > 5.1 [18]. Boolean remission requires swollen and tender joint counts, C-reactive protein (CRP in mg/dl), and patient global assessment (PGA on a 0–10 scale) to be ≤ 1 [17].

### Statistical analysis

We performed statistical analysis with SPSS [version 25] and expressed patient characteristics as means with standard deviation (SD) or median with inner quartiles as appropriate. We assessed the correlation between the RAPID3 and DAS28 with the Spearman’s rank correlation coefficient and calculated the agreement with Cohen’s kappa classified as no agreement (< 0), slight (0–0.20), fair (0.21–0.40), moderate (0.41–0.60), substantial (0.61–0.80), and almost perfect agreement (0.81–1.0) [19]. We calculated the agreement for four categories of disease activity (remission, low, moderate, and high) with a weighted kappa. To determine the best RAPID3 cut-off point for low versus high disease activity, we constructed a receiver-operating characteristics (ROC) curve. We considered DAS28 scores below 3.2 as “low” and 3.2 and higher as high. In addition, we calculated the sensitivity, specificity, and positive predictive value (PPV) of the RAPID3 to test for high or low disease status (as determined by a high or low DAS28). Also, the percentage of patients with a RAPID3 below 2.0 was calculated to help determine the best cut-off point. Furthermore, RAPID3 scores were not only used to test for DAS low disease activity but also for Boolean remission. Initially, we regarded the assessment pairs as independent observations, even though some patients provided data on multiple occasions. In order to assess if the results were biased due to this, we performed a sensitivity analysis with only the first measurement of each patient.

### Ethics

We performed this study using available clinical patient records of Reade. All collected data were anonymized. The procedures in this investigation were in accordance with legislation (the Medical Research Involving Human Subjects Act) and ethical standards on human experimentation in the Netherlands. After consultation with the Medical Ethics Review Committee (METc) of the Amsterdam University Medical Centre location Vrije Universiteit and the local data protection officer of Reade, the requirement for informed consent was waived since we used retrospective anonymous data collected as part of usual care.

## Results

### Patient characteristics

A total of 5009 combined RAPID3 and DAS28 measurements were performed at Reade between June 2014 and March 2021 on a total of 1681 unique RA patients. A total of 587 patients provided one, 367 two, 232 three, and 495 four or more assessment pairs. Patient demographics were as expected, with mostly female patients and seropositive RA (Table 1).

**Table 1:** Patient characteristics

Total measurements	5009
Total patients	1681
Female (%)	1270 (76)
Age, mean (SD)	60 (14)
Disease duration, median (1st–3rd quartiles)	4 (1–7)
Type of RA, *n* = 1146 (%)	
RF-positive	89 (9)
aCCP-positive	211 (18)
Both negative	267 (23)
Both positive	575 (50)
DAS28, median (1st–3rd quartiles)	2.6 (1.8–3.6)
DAS28 per category (%)	
Remission	2477 (49)
Low	837 (17)
Medium	1356 (27)
High	339 (7)
RAPID3, median (1st–3rd quartiles)	3.7 (1.8–5.5)
RAPID3 per category (%)	
Remission	735 (15)
Low	691 (14)
Medium	1284 (26)
High	2299 (46)
RAPID3 components, median (1st–3rd quartiles)	
Function	2.7 (1.3–4.3)
Pain	4.0 (1.5–6.5)
PGA	4.5 (2–6.5)
CRP, median (1st–3rd quartiles)	2.4 (0.9–7.7)
ESR, median (1st–3rd quartiles)	9 (5–24)

*SD*, standard deviation; *RA*, rheumatoid arthritis; *RF*, rheumatoid factor; *aCCP*, anti-cyclic citrullinated peptide antibody; *DAS28*, disease activity scale; *RAPID3*, Routine Assessment of Patient Index Data 3; *PGA*, patient global estimate of status; *CRP*, C-reactive protein; *ESR*, erythrocyte sedimentation rate

### Correlation and agreement between RAPID3 and DAS28

The (rank) correlation between RAPID3 and DAS28 was 0.58 for all records and 0.57 (both *p* < 0.001) for the first measurement of each patient. The agreement was fair for both the four categories of disease activity and the binary high/low categories (Tables 2 and 3). Results were very similar when the analysis was repeated on only the first measurement of individual patients (weighted kappa = 0.21). In total, 28% of the patients were classified as having low disease activity. When the RAPID3 classified the patient as low, 92% of the cases were also in a DAS28 in the low category. However, when the RAPID3 classified a patient in the high category, the DAS28 corresponded in 44% of the cases. The sensitivity analysis yielded similar results, 90% of the cases that were classified into the low disease activity by the rapid 3 were also classified into the low category of the DAS28.

**Table 2:** Cross tabulation of RAPID3 and DAS28 results by category of disease activity

		**DAS28**				Total
		**Remission (< 2.6)**	**Low (2.6–3.2)**	**Moderate (3.2–5.1)**	**High (> 5.1)**	
**RAPID3**	**Remission (≤ 1.0)**	642 (13%)	49 (1%)	40 (1%)	4 (< 1%)	735 (15%)
	**Low (1.1–2.0)**	503 (10%)	115 (2%)	70 (1%)	3 (< 1%)	691 (14%)
	**Moderate (2.1–4.0)**	704 (14%)	281 (6%)	275 (5%)	24 (< 1%)	1284 (25%)
	**High (≥ 4.0)**	628 (13%)	392 (8%)	971 (19%)	308 (6%)	2299 (46%)
**Total**		2477 (49%)	837 (17%)	1356 (27%)	339 (7%)	5009 (100%)

^*^Percentage agreement: 1340/5009 = 27%. Weighted kappa: 0.21. Listed percentages refer to the grand total

**Table 3:** Cross tabulation of RAPID3 and DAS28 results by binary categories of disease activity

		**DAS28**		**Total**
		**Low (< 3,2)**	**High (> 3,2)**	
**RAPID3**	**Low (≤ 2.0)**	1309 (26%)	117 (2%)	1426 (28%)
	**High (> 2.1)**	2005 (40%)	1578 (32%)	3583 (72%)
**Total**		3314 (66%)	1695 (34%)	5009 (100%)

DAS28 low =  < 3.2, DAS28 high =  ≥ 3.2, RAPID3 low =  ≤ 2.0, RAPID3 high =  > 2.0* Percentage agreement: 58%. Kappa: 0.26

#### RAPID3’s cut-off for low disease activity

**Fig. 1:**
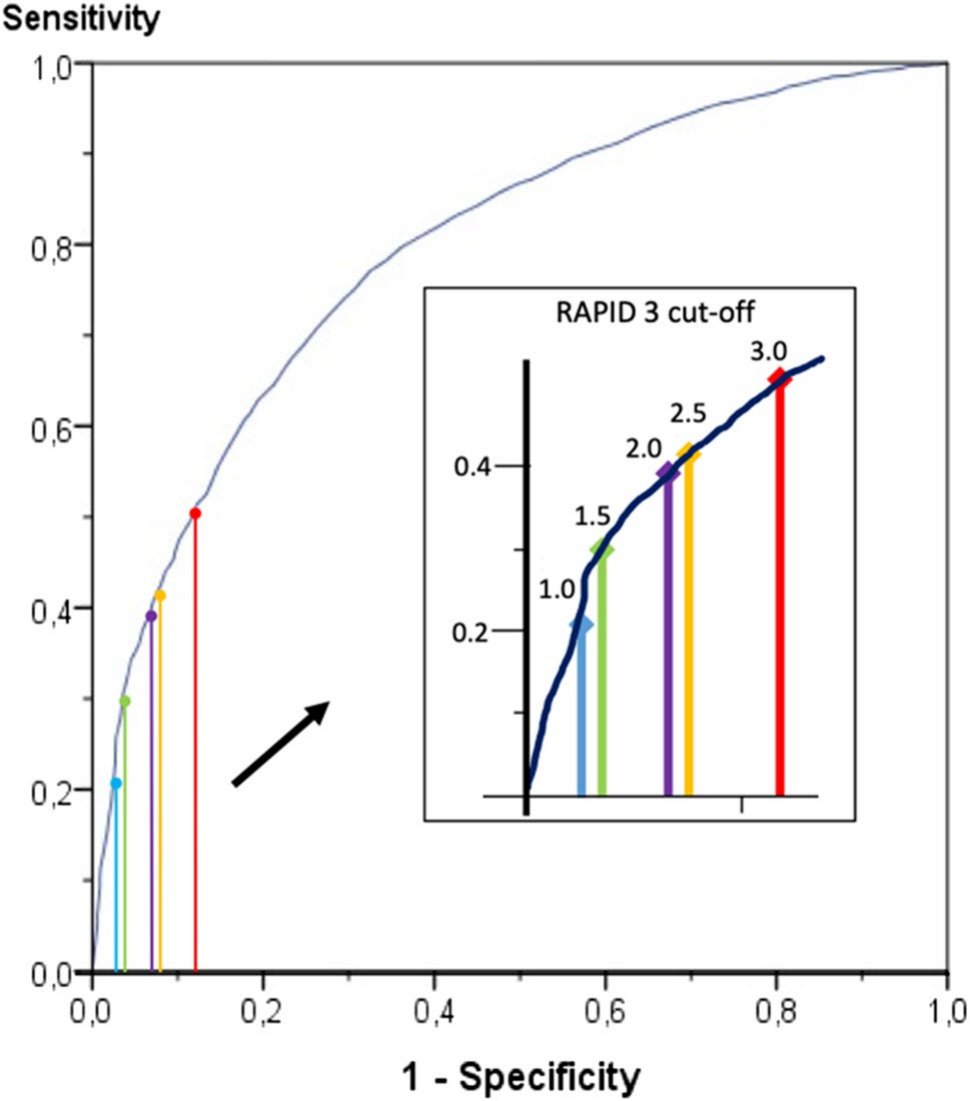
ROC curve of RAPID3 scores. Receiver operating characteristic (ROC)

We constructed a receiver operating characteristic (ROC) curve to determine the best RAPID3 cut-off point for low disease activity, see Fig. 1. The best cut-off point was the recommended value, i.e., 2,0, based on the sensitivity and specificity. The area under the curve is 0.80 (95% CI: 0.78–0.81). Table 4 shows the sensitivity, specificity and PPV for different RAPID3 cut-off points. We performed an in-depth analysis of the cases in which the RAPID3 shows low disease activity but according to the DAS28 high to investigate which aspect of the DAS28 was responsible for this difference in classification, see Table 5.

**Table 4:** Sensitivity, specificity, positive predictive value, and % of patients classified as low disease activity corresponding to the ROC curve

Cut-off value RAPID3	Sensitivity	Specificity	PPV	% patients classified in remission/low
1.0	0.21	0.97	0.94	15%
1.5	0.30	0.97	0.94	16%
2.0	0.39	0.93	0.92	28%
2.5	0.41	0.91	0.91	30%
3.0	0.54	0.86	0.88	41%

**Table 5:** In-depth analysis of the cases with a RAPID3 score classified as low, but according to the DAS28 high, for three different RAPID3 cut-off points: 1.0, 1.5, and 2.0

	**RAPID3 ≤ 1.0**	**RAPID3 ≤ 1.5**	**RAPID3 ≤ 2.0**
Total cases	735	1077	1426
Cases with DAS28 > 3.2 (%)	44 (6%)	64 (6%)	117 (8%)
DAS28 median (IQR)	3.71 (3.3–4.2)	3.6 (3.3–4.2)	3.61 (0.73)
DAS28 cat			
Moderate	40	58	110
High	4	6	7
ESR median (IQR)	29 (19–51) (*n* = 28)	27 (19–52) (*n* = 44)	27 (16.0–44.0) (*n* = 81)
TJC* median (IQR)	2 (1–4)	2 (1–4)	2 (1–3)
0	7 (16%)	15 (23%)	19 (16%)
1	12 (27%)	13 (20%)	27 (23%)
2	8 (18%)	12 (19%)	29 (25%)
3	5 (11%)	7 (11%)	14 (12%)
4	2 (5%)	2 (3%)	6 (5%)
> 4	10 (23%)	15 (23%)	22 (19%)
SJC* median (IQR)	2 (1–4)	2 (1–4)	2 (1–4)
0	6 (16%)	11 (17%)	22 (19%)
1	12 (25.0%)	17 (27%)	31 (27%)
2	5 (13.6%)	8 (13%)	17 (15%)
3	5 (9.1%)	9 (14%)	12 (10%)
4	3 (4.5%)	5 (8%)	12 (10%)
> 4	14 (23%)	14 (22%)	23 (20%)
DAS28 patient general health mean (SD)	30 (28.3) (*n* = 41)	31 (27) (*n* = 61)	28.1 (22.8) (*n* = 108)
RAPID3 PGA* mean (SD)	1.2 (2.2) (*n* = 40)	1.1 (1.8) (*n* = 57)	1.4 (1.6) (*n* = 106)

^*^TJC, tender joint count; *SJC*, swollen joint count; *PGES*, patient general estimate of status

### Boolean remission

In 1153 cases, the required variables were present to calculate Boolean remission. Boolean remission was present in 122 of 1153 cases (11%). The sensitivity of the RAPID3 to detect Boolean remission was 0.80, the specificity 0.68, the negative predictive value 0.97, and the positive predictive value 0.23.

## Discussion

This study illustrates that 28% of clinic visits could be postponed with a RAPID3 cut-off point of 2.0; in 2% of these (8% of the 28%), the underlying DAS28 would be above 3.2, and postponement could be regarded as unjustified. In these, slightly less than half had no or only one painful or swollen joint. This indicates that the concept of prescreening is feasible but also that the RAPID3 has some limitations as a screening tool.

Our results confirm that RAPID 3 tends to overestimate disease activity compared with the DAS28 [7]. According to the DAS28 scores, 66% of the patients are in a low disease activity state, compared to only 28% according to the RAPID3. Furthermore, 13% of the patients that are classified in the highest disease state by the RAPID3 are classified in the lowest disease state by the DAS28. In contrast, the in-depth analyses of the cases with a low RAPID3 but high DAS28 showed that even in patients with very low RAPID3 scores, some have swelling of multiple joints. In fact, 23% of the misclassified patients with a RAPID3 below 1.0 had over 4 swollen joints. Possible explanations could be that (1) there is a discordance between what patients feel and what is observed, (2) the RAPID3 is sometimes misinterpreted by patients, or (3) that the RAPID3 value fluctuates significantly from day to day*.* The overestimation of disease activity, combined with the acceptable number of patients that report low RAPID3 scores, makes the RAPID3 particularly useful to screen for DAS low disease activity. However, following the Boolean criteria, only 11% of the patients are in remission. The low number of patients in Boolean remission, combined with the low positive predictive value indicate that screening for Boolean remission with the RAPID3 is not feasible. Our results also confirm that DAS28 “remission” does not correspond with Boolean remission, but is better termed “minimal disease activity [20].”

This is the first study that focuses on the accuracy of the RAPID3 to identify low disease activity according to the DAS28. Therefore, there is no readily available literature to compare our results with. However, previous research did evaluate the agreement between the two scores, allowing us to perform similar analyses in order to compare our results. Eight studies showed a wide range in sensitivity (ranging from 0.39 to 0.88), specificity (0.73 to 0.96), and PPV (0.50 to 0.91) to identify low disease activity [8–15]. The best study for comparison analyzed routine care data of a clinical routine outpatient clinic in the Netherlands [8]. In this study, sensitivity was 0.40, specificity 0.96, and the PPV 0.91. The heterogeneity in the other studies may be partially explained by cultural or ethnic differences in illness perceptions and pain attitudes as described in a recent systematic review [21] and the trial setting, whereas our study was based on routine care data. Thus, our results are generalizable to routine care, specifically in the Netherlands or countries with similar illness perceptions and pain attitudes. Regarding other PROs, Mistry et al. recently compared the RAID with the DAS28 [22]. In 218 observations in the UK, they found a slightly better PPV of 0.98 with a DAS28 cut-off at < 3.2 and a similar proportion of patients eligible for postponement (30%). However, when the desired DAS28 is set at < 2.6, the PPV remained high for the RAID (0.92), whereas the RAPID3 decreased to 0.80. A comparison between different PROs should determine which PRO is most suitable for screening for low disease activity.

Ideally, a patient-reported algorithm (or sequence of questions) could be developed that consists of a little questions as possible with an as high as possible accuracy. We propose a system in which an outpatient clinic visit is postponed/omitted when the RAPID3 is 2.0 or lower. However, a telephone consultation may be needed when a patient wants to speak with the rheumatologist despite the low RAPID3 or when elevated laboratory parameters in the periodic screening for side effects of medication in blood samples demand patient-rheumatologist interaction.

### Limitations

This study has several limitations. First, retrospective studies are susceptible to confounding and selection bias. In terms of selection bias, the study analyzed only the results of patients with complete questionnaires and a DAS28. It could be that our group had above-average self-management or eHealth literacy as compared to the general population. Also, patients excluded for incomplete records (DAS28 or RAPID3) are more likely to have low disease activity as the impetus to measure is higher in patients suspected of having high disease activity. However, given the above-cited studies, we believe our results to be generalizable to patients in the Netherlands. Second, the DAS28 as a “gold standard” has limitations of its own. Originally developed to quantify RA disease activity in clinical trials, it has several flaws in the evaluation of individual patients. These include underestimation of the disease activity as the joints of the feet are not measured, overestimation due to elevated ESR associated with non-rheumatologic conditions, and unreliable joint counts [23]. These flaws could lead to a misclassification of remission or low disease activity by the DAS28, and therefore patients with a low RAPID3 (and consequently a low DAS28) might still have some chance of not being in remission. Comparing the RAPID3 with the Boolean criteria for remission was difficult due to the low number of patients classified in Boolean remission. This is in line with recently performed studies, finding that Boolean remission in clinical practice is hard to achieve [24, 25]. By comparing the RAPID3 with the frequently used DAS28, we think that the results have more value for clinical practice. Thirdly, since this is a cross-sectional study, we were unable to determine if the state of low disease activity remained over time, or that the disease activity flares in the months after the RAPID3 measurement. To investigate this, future longitudinal studies are necessarily focused on the period after the reported RAPID3 before the implementation of our proposed system.

## Conclusion

In 92% of the cases where patients score a RAPID3 of ≤ 2.0, the DAS28 is ≤ 3.2. In our routine, care approximately a quarter of assessments are at or below this level and could potentially be postponed. We propose a system where consultations are postponed when the RAPID3 is ≤ 2.0 to reduce the number of outpatient clinic visits for patients with low disease activity. Such a proposal is safe if the patient can overrule it.
